# Aging’s Effect on Working Memory—Modality Comparison

**DOI:** 10.3390/biomedicines12040835

**Published:** 2024-04-10

**Authors:** Eyal Heled, Ohad Levi

**Affiliations:** 1 Department of Psychology, Ariel University, Ariel 4077625, Israel; levi1ohad1i@gmail.com; 2 Department of Neurological Rehabilitation, Sheba Medical Center, Ramat Gan 5262160, Israel

**Keywords:** working memory, Tactual Span, Digit Span, Visuospatial Span, aging, development

## Abstract

Research exploring the impact of development and aging on working memory (WM) has primarily concentrated on visual and verbal domains, with limited attention paid to the tactile modality. The current study sought to evaluate WM encompassing storage and manipulation across these three modalities, spanning from childhood to old age. The study included 134 participants, divided into four age groups: 7–8, 11–12, 25–35, and 60–69. Each participant completed the Visuospatial Span, Digit Span, and Tactual Span, with forward and backward recall. The findings demonstrated a consistent trend in both forward and backward stages. Performance improved until young adulthood, progressively diminishing with advancing age. In the forward stage, the Tactual Span performance was worse than that of the Digit and Visuospatial Span for all participants. In the backward stage, the Visuospatial Span outperformed the Digit and Tactual Span across all age groups. Furthermore, the Tactual Span backward recall exhibited significantly poorer performance than the other modalities, primarily in the youngest and oldest age groups. In conclusion, age impacts WM differently across modalities, with tactile storage capacity being the most vulnerable. Additionally, tactile manipulation skills develop later in childhood but deteriorate sooner in adulthood, indicating a distinct component within tactile WM.

## 1. Introduction

Working memory (WM) is the ability to store information for a short period of time and manipulate it to achieve a specific goal [1,2,3]. This is a crucial ability for proper functioning throughout the lifespan, as it is closely linked to a variety of cognitive abilities, including reasoning, attention, language comprehension, reading, and abstract thinking [4,5,6,7].

One of the central models to describe the structure and function of WM is the multicomponent model [4,8]. According to the latter, WM is divided into four modules. The first is the central executive, which is responsible for manipulating information stored for short terms by two other modality-based storage modules of verbal and visuospatial nature: the phonological loop and the visuospatial sketch pad, respectively [9]. Later, another module, named the episodic buffer, was added, which is responsible for integrating information from several sources, including long-term episodic memory [8].

A particular aspect of research on WM has focused on developmental and aging processes, revealing that WM is employed from early childhood and then continues to improve into young adulthood [10,11]. Moreover, studies suggest that the underlying structure of WM is established by ages 4–6 [10,12] and shows constant improvement through adolescence and young adulthood. However, the literature is inconsistent about when this improvement ends. Moderate declines have been noted at age 26 [11] and 40 [13], though another study showed no decline at all at these ages [14]. Further, studies have shown that the decline in WM does not occur uniformly, suggesting either a more rapid deterioration around the age of 40 [11], 60 [14,15,16], or no accelerated decline in old age; rather, there is a steady and moderate decay [13,17,18,19]. Although there is no consensus on the age of onset and rate of decline, all studies have shown that age has some level of effect on WM.

Interestingly, the vast majority of studies that have investigated age effects on WM modalities almost exclusively focused on the verbal and visuospatial modalities, showing consistent deterioration from young to older adulthood, although the relation between the modalities was not clear [11,17,18,19,20]. On the one hand, it was found that children reach a maximum capacity of visual WM at an earlier age than verbal WM, thus beginning to deteriorate sooner—around adolescence [11,21]. Another study compared young adults aged 18–22 to elderly participants of 63–69 on verbal and visuospatial WM and found that the ability to recall verbal information better than the ability to recall visuospatial information. However, the difference was more prominent in the elderly group, indicating an interaction effect between age and WM modality [22]. Similarly, comparing a young adult group to older adults aged 62–77 has shown that visuospatial WM is more susceptible to age than verbal WM [23], and a cross-sectional study from childhood to age 59 showed poor visuospatial WM in old age compared to verbal WM [17]. Such a decline in visuospatial, compared to verbal, WM in older adults was accounted for by the right hemi-aging model, which suggests that aging induces an increased decline in the right hemisphere, involved in visuospatial information processing, compared to the left hemisphere, which is associated with verbal skills [24].

On the other hand, some authors found a different performance pattern, showing no difference between visuospatial and verbal WM across the ages of 20 to 70 [13] and 20 to 80 [19]. Kumar and Priyadarshi [18] compared verbal and visuospatial WM storage and manipulation in five age groups of 40 to above 80, revealing a consistent performance decrease, with no significant difference between them.

Nevertheless, while much attention has been paid to the verbal and visuospatial modalities, the effect of age on tactile WM has been investigated to a far lesser extent, probably because of its relatively reduced use in everyday functioning [25]. Tactile WM is defined as the ability to store and process tactile information for short time periods [26], and several studies have shown that its capacity is significantly more limited compared to the verbal and visuospatial modalities [27,28,29].

A literature search revealed almost no studies that examined tactile WM across different ages, and even fewer compared it to verbal and visuospatial modalities. One such example showed that WM performance of storage and manipulation separately improved over age in all modalities, although the tactile modality showed the lowest performance compared to the visuospatial, visual, and verbal modalities [30]. Another study showed similar findings when examining only storage ability [31]. Therefore, it was concluded that tactile WM has the same development course as other modalities, but shows much lower capacity.

In light of the evidence indicating the inconsistency of WM ability in different modalities across age groups, as well as the scarce knowledge on the tactile modality, including in comparison to other modalities, the current study aimed to evaluate the effect of age (i.e., childhood, young adulthood, and elderly) on WM functions in terms of both storage and manipulation in different modalities (i.e., verbal, visuospatial and tactile). Our findings could shed light on age-related aspects of WM ability across modalities, specifically the tactile one. Thus, this study could contribute to distinguishing between normal and pathological conditions.

## 2. Materials and Methods

### 2.1. Participants

We utilized the G*Power analysis software (Version 3.1.9.7, Heinrich-Heine-Universität Düsseldorf, Düsseldorf, Germany [32]) to determine the appropriate sample size, which initially indicated a minimum of 105 participants. However, in order to enhance the statistical power of the study, we increased the sample size beyond this minimum requirement. One hundred thirty-four participants took part in the study (52 girls and women) who were divided into four age groups in the range of 7–69. The first group included 32 children aged 7–8; the second group contained 32 children aged 11–12 (previously presented in Heled et al., 2022 [30]); the third group was composed of 36 young adults aged 25–35; and the fourth group included 34 participants in the age range of 60–69. The children who volunteered to participate in the study were obtained from class chat groups of the parents who approved their participation, and they were not rewarded. The young adults were mostly undergraduates who participated in the study to meet course requirements and were obtained from social media, and word of mouth. Recruitment of the older-age group occurred through a network of nursing homes, whose management granted approval for the research and permitted the invitation of volunteers to their weekly meetings. They received a modest gift for their participation. Exclusion criteria for the study included: (a) a history of psychiatric or neurological disorders (e.g., epilepsy, ADHD), (b) the existence of a sensory or motor impairment that did not enable computer use, and (c) a diagnosis of a learning disability or developmental disorder. Inclusion criteria included: (a) right-hand dominance and (b) for adults, between 12 and 16 years of education. The Ethics Committee of Ariel University approved the study (AU-EH-20181220).

### 2.2. Instruments and Measures

#### 2.2.1. Demographic and Clinical Questionnaire

A self-report questionnaire was created for the study and included demographic information (e.g., age, sex, and years of education).

#### 2.2.2. Tactual Span

The Tactual Span task evaluates WM in the tactile modality [28]. In this task, the participant is asked to place four fingers of each hand on a row of computer keyboard keys and cover his/her eyes. Then, the experimenter begins the forward stage by sequentially touching the knuckles of the participant’s fingers for 1 s each in a predetermined order. The participant is asked to repeat this sequence by pressing the corresponding keys on the keyboard with the same fingers that were touched. Each sequence length consists of three trials, starting with two fingers and then increasing in one stimulus if the participant is correct in at least one trial. The maximum sequence length is nine stimuli. If the participant fails all three trials of the same sequence, the task is stopped and then continues to the backward stage. In this stage, the experimenter touches the knuckles of the participant’s fingers in a predetermined order, but now the participant is instructed to press the keys in reverse order. The dependent variable is the longest sequence recalled by the participant for the backward and forward stages separately.

#### 2.2.3. Digit Span

The task was taken from the Wechsler Adult Intelligence test—third edition [33] and was computerized. In the first stage—forward recall, a series of numbers ranging from 1 to 9 are presented orally for 1 s per stimulus, and the participant is then asked to repeat them orally in the same order presented. The sequences begin by presenting two auditory stimuli, with a single digit added to each sequence when the participant is correct, until they are unable to recall two trials of the same length. In the second backward stage, the procedure is the same, but the participant is required to state the numbers in reverse order. The dependent variable is the longest sequence the participant could recall in each stage.

#### 2.2.4. Visuospatial Span

The Visuospatial Span task is a computerized version of the Corsi Block-Tapping Test [34], to evaluate WM in the visuospatial modality. Nine purple squares are presented on the screen in a mixed array. The squares change color to yellow, one by one, for 1 s each in a predetermined pseudo-random order. The participant is then asked to use the mouse and click on the squares in the same order they changed color (i.e., forward stage) beginning in two trials of two squares, and if at least one is correct, another square is added to the sequence. This continues until the participant fails two trials of the same length. The procedure remains the same in the second—backward stage, but now the participant is required to repeat the squares in reverse order. The longest sequence score is the dependent variable, assessed separately for the forward and backward stages.

### 2.3. Procedure

After receiving a brief explanation of the study, all participants (or legal guardians) signed an informed consent form and completed the demographic questionnaire. Next, participants undertook the experiment, which lasted about an hour.

### 2.4. Statistical Analysis

The groups were compared in terms of age and education using a one-way analysis of variance (ANOVA) for each variable, followed by a Bonferroni post hoc test. Concerning gender, a chi-square test was used to compare the number of males and females. Then, all age groups across all span tasks were compared by applying a 3 (Task) × 4 (Group) repeated-measures ANOVA in each stage (i.e., forward and backward). This analysis was followed by a Bonferroni post hoc test to assess the source of the difference. In cases of an interaction effect, we used an ANOVA test to assess simple effects between the modalities in each age group. Data were analyzed using SPSS version 27, and alpha was determined at *p* < 0.05.

## 3. Results

First, a comparison of the four age groups showed a significant difference (*F*(3,133) = 4416.9, *p* < 0.001, η_p_^2^ = 0.99). Comparing the groups by years of education also demonstrated a significant difference (*F*(3,133) = 630.13, *p* < 0.001, partial η_p_^2^ = 0.93), which was shown to be between the children’s and adult groups. Comparing the number of males and females between the groups yielded a non-significant result (χ^2^(3) = 0.599, *p* = 0.112; see Table 1).

**Figure 1:**
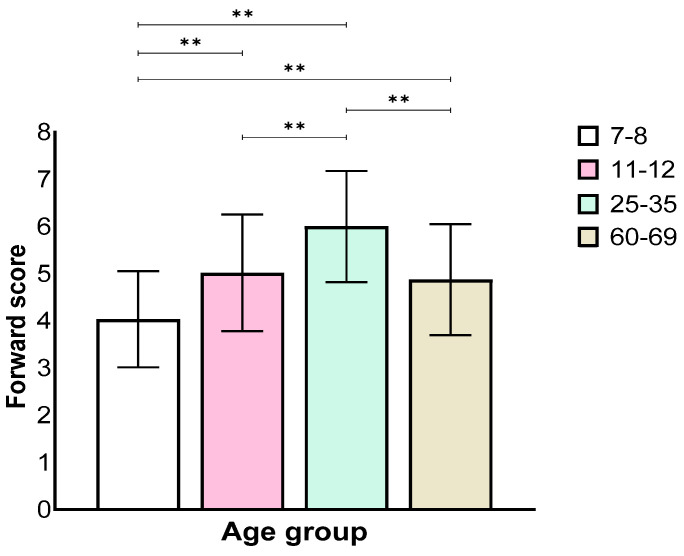
Means and standard deviations of the four age groups’ longest sequence score in the forward stage. ** *p* < 0.001.

Analysis of the forward stage showed a main effect for the age group (*F*(3,129) = 34.21, *p* < 0.001, η_p_^2^ = 0.44). Further, a Bonferroni post hoc test revealed worse performance of the 7–8 age group when compared with all other groups, and the 11–12 age group performed worse than the 25–35 age group, but not the 60–69 age group. The 25–35 age group performed significantly better than the other groups (Figure 1).

**Figure 2:**
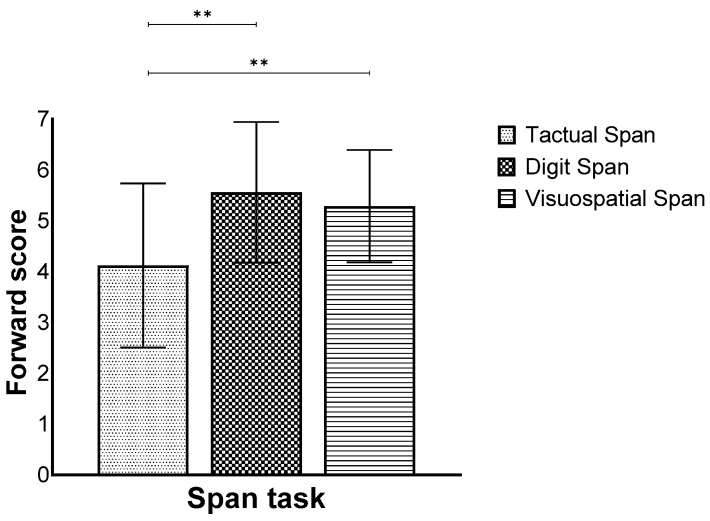
Means and standard deviations of the span tasks’ longest sequence score in the forward stage by modality. ** *p* < 0.001.

Furthermore, we found a main effect for modality (*F*(2,258) = 68.82, *p* < 0.001, η_p_^2^ = 0.34), while the post hoc test showed that the Tactual Span was performed worse compared to the other span tasks (Figure 2).

**Figure 3:**
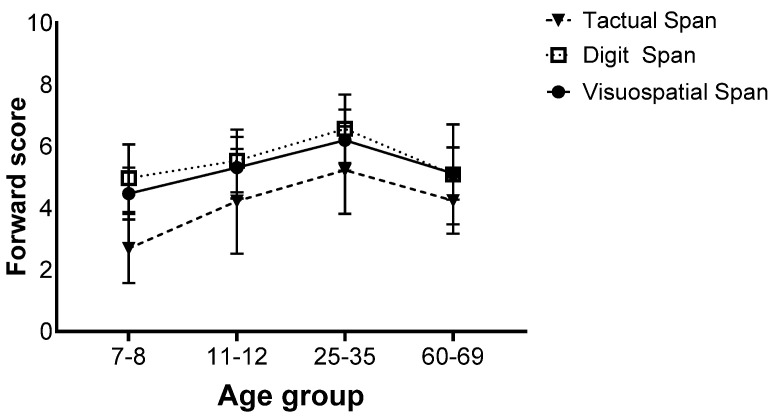
Means of the span tasks’ longest sequence scores and standard deviations in the forward stage of the four age groups.

There was also an interaction effect (*F*(9,258) = 2.75, *p* = 0.013, η_p_^2^ = 0.06), and further analyses revealed that for all age groups, the Tactual Span was performed worse than the Digit and Visuospatial Span. No difference was found between the Digit and Visuospatial Span (Figure 3).

**Figure 4:**
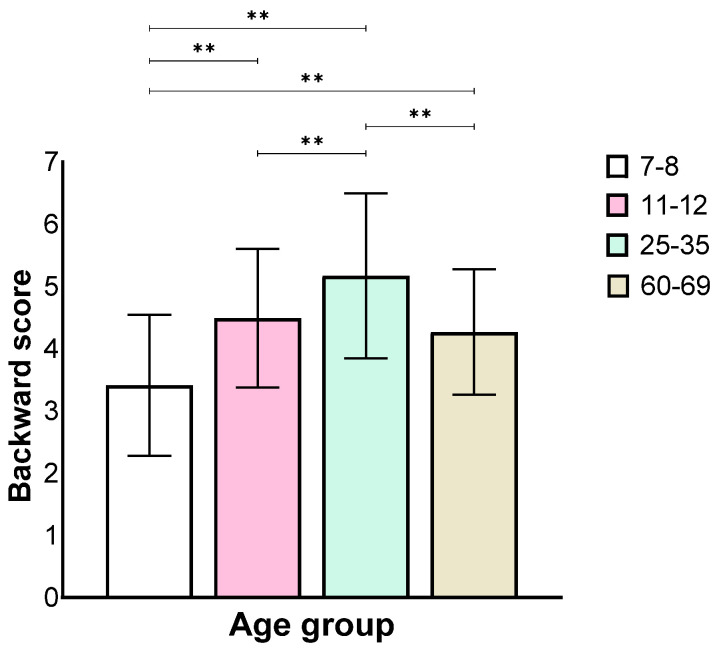
Means and standard deviations of the four age groups’ longest sequence score in the backward stage, ** p < 0.001.

Analysis of the backward stage showed a main effect for the age group (*F*(3,130) = 27.84, *p* < 0.001, η_p_^2^ = 0.39), similar to the forward stage, where the 7–8 age group performed worse than all the other groups and the 25–35 age group performed better than all the other groups. There was no difference between the 11–12 and the 60–69 age groups (Figure 4).

**Figure 5:**
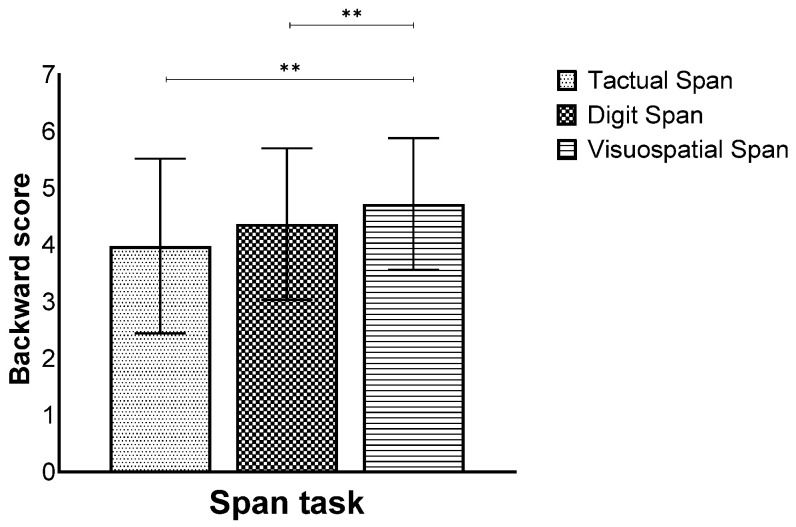
Means and standard deviations of the span tasks’ longest sequence score in the backward stage by modality, ** p < 0.001.

In addition, a main effect for modality was also found (*F*(2,260) = 17.55, *p* < 0.001, η_p_^2^ = 0.11), showing that the Visuospatial Span was performed better than the Tactual and Digit Span (Figure 5).

**Figure 6:**
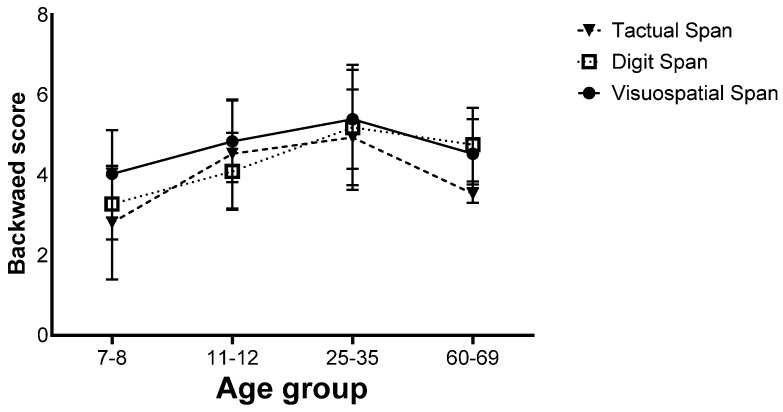
Means of the span tasks’ longest sequence scores and their standard deviations in the backward stage of the four age groups.

Finally, there was a significant interaction effect (*F*(6,260) = 4.66, *p* < 0.001, η_p_^2^ = 0.09). Further analyses showed that in the 7–8 age group, the Visuospatial Span was performed better than the Digit and Tactual Span, and in the 11–12 age group, the Visuospatial Span was performed better than the Digit Span, but in the 25–35 age group, no difference was found between the different tasks. In the 60–69 age group, the Tactual Span was performed worse than the Visuospatial and Digit Span (Figure 6).

## 4. Discussion

The purpose of the current study was to compare the WM capacity of storage and manipulation across different modalities (i.e., verbal, visuospatial, and tactile) in four age groups: 7–8, 11–12, 25–35, and 60–69. We found that for both the forward and backward stages of all tasks, there was an improvement in performance until young adulthood, followed by a decline in old age. There was also a main effect for modality in both stages, demonstrating poor performance in the Tactual Span compared to the Digit and Visuospatial Span. In addition, performance in the backward stage of the Visuospatial Span was better than that of the Digit and Tactual Span in all groups. Further, we found an interaction effect for both stages, showing that in the forward stage, the Tactual Span was performed worse than the Digit and Visuospatial Span in all age groups. In the backward stage, the 7–8 age group performed the Visuospatial Span better than the Digit and Tactual Span, the 11–12 age group performed the Visuospatial Span better than the Digit Span, and the 60–69 age group performed the Tactual Span worse than the Visuospatial and Digit Span.

Improvement in WM capacity until adulthood, as was found in the current study, is supported in the literature on WM development in the verbal and visuospatial modalities [17,22,35,36,37,38] as well as the tactile modality [30,31]. Skills development in rehearsing information, improved storage capacity, cognitive abilities, and strategy use [15,17,38,39,40] have been suggested to explain this trend. Neurological changes in synapse connectivity, white matter density, and brain area specialization in charge of processing the information were also suggested to account for WM improvement [41,42,43,44]. Therefore, the increase in cognitive and neurological processes occurs simultaneously throughout childhood and adolescence [21].

However, with brain aging, the opposite process ensues where there is cortical atrophy, age-related reduction in global blood flow, and reduction in brain function [45]. Consequently, WM performance deteriorates and performance is poorer among older, as compared to younger adults [11,15,22,45,46,47].

In the present study, performance in the tactile modality was worse as compared to other modalities in the storage and manipulation components, most likely because tactile-related functions are much less used in daily functions than other modalities [25,48]. However, it is unclear whether there are additional factors, like the neurological pre-disposition of tactile low capacity, that contribute to poorer performance. Studies among sensory-deprived participants [49] found that people with deafness demonstrated better visuospatial WM but equal tactile WM, and people with blindness demonstrated better auditory and tactile WM when compared to persons with no hearing or visual impairment. These results support the suggestion that everyday practice may have the most impact on modality-related WM performance [49,50].

In the backward stage, participants exhibited superior performance on the Visuospatial Span compared to the Digit Span, and their performance on the Digit Span was better than on the Tactual Span. These findings indicate a notable differentiation between modalities in terms of manipulation ability [22]. Such differentiation in the backward but not the forward stage may be attributed to differences in aging processes among modalities that influence performance. Namely, the executive component of WM is more sensitive to age than the storage component. Indeed, in a study that examined the executive component of WM (i.e., updating) across adults’ life span, verbal WM deteriorated sooner than visuospatial WM, but the visuospatial decline was steeper than verbal WM [20]. The authors concluded that WM modality is affected by age and that verbal skills are much more developed than visuospatial skills and, therefore, decay slower. Thus, aging has a differentiated effect on modality-related manipulation [11,17,23].

In line with this, the interaction effects corroborate the view that storage and manipulation components should be understood in the context of a differentiation between modalities and WM components as we age [17]. In the forward stage, we found that only the Tactual Span was performed less well than the Visuospatial and Digit Span, but no difference was found between the Digit and Visuospatial Span. Therefore, we may conclude that the tactile modality has the lowest storage capacity of all abilities regardless of age, and that, by the age of 7, the ability to store information is similar for verbal and visuospatial modalities. This pattern seems to remain stable throughout young adulthood and old age. Other studies have also found that visuospatial and verbal WM decay at comparable rates [13,19], thus indicating that neurological and cognitive factors known to influence storage, decline equally in both modalities. However, these results contrast with others that found a visuospatial tendency to deteriorate faster than verbal storage [23]. Therefore, this should be further investigated.

Nevertheless, as opposed to the forward stage, the backward stage displayed an inconsistent pattern, suggesting that the two task stages assess distinct abilities [51,52]. The Visuospatial Span was performed better than the Digit Span among the younger groups, similar to several other studies, which suggested language skills are not yet well acquired at early elementary school age; therefore, the visuospatial modality might compensate for this [53,54]. Although this suggestion may be relevant to the youngest age group (i.e., 7–8), by ages 11–12, language skills are already established; therefore, a more logical explanation may be that verbal manipulation is more cognitively demanding in this age range than visuospatial manipulation, making the performance of the verbal modality poorer than the tactile and visuospatial modalities [54].

Regarding the Tactual Span, it seems that there is an inconsistent trend, whereby a difference was found among 7- and 8-year-olds but not in the 11–12 or 20–29 age groups. This may be explained by the increasing use of digital devices that demand using the hands and have an impact on different executive tactile-related functions, including WM [55,56]. Furthermore, because the Tactual Span tasks were performed worse only in the youngest and oldest groups, it may show that tactile manipulation has a dynamic character compared to the other modalities, such that it is developed later and decays sooner in life due to everyday use or brain functions. This issue should be further elucidated.

The distinction between verbal and visuospatial WM at different ages may be attributed to brain function changes, as it was found that there is an earlier decrease in visuospatial functions that are associated with the right hemisphere, compared to verbal functions underpinned by the left hemisphere [24]. However, while studies showed this trend in visuospatial and verbal WM, the current study indicated that this is also true for the tactile modality in both the storage and manipulation components. We did not find studies that compared young and old age groups in tactile WM and other modalities, making this line of research innovative.

There were several limitations to the present study. First, it is a cross-sectional study and, as such, is limited in concluding individual changes in WM of different modalities. Moreover, the findings could be better generalizable if the sample size was larger. Third, there was no consistency in the reward given to the participants, such that some of the young adults participated as part of course requirements, the older adults received a modest gift in exchange for their participation, and others from the young adults group and the two children’s groups did not benefit from the study. Fourth, the fact that we merged data across different studies, particularly for the younger age groups, may potentially introduce a bias due to variations in experimental conditions, timing, equipment, and recruitment methods.

Future studies should elaborate on the number of age groups examined in children and adults. Following findings on the influence of other cognitive abilities on WM performance [13,19], further research could combine other cognitive tasks, such as those related to inhibition and processing speed. Finally, it would be interesting to investigate Baddeley’s model concerning its structure of standalone tactile storage [26,57] or as part of the visuospatial sketchpad [4].

In conclusion, our study reveals that the development and aging process of WM shows the same performance pattern between different modalities. Namely, in the age range of childhood to young adulthood, there is an improvement in WM, but in old age, there is a decline in performance, indicating that age affects WM capacity. In addition, the tactile modality in the storage and manipulation components is mostly performed worse than the other WM modalities, thus essentially being the most demanding modality of the three. This was true for each age group in the storage ability but only for the youngest and oldest groups in the manipulation ability, suggesting that tactile WM develops later in childhood and deteriorates sooner in adulthood. Consequently, our results may point to a distinct component for the tactile modality in WM, differentiated from verbal and visuospatial modalities.

## Figures and Tables

**Table 1:** Means and standard deviations (in parentheses) of the participants’ age and education years divided into the age groups.

	7–8	11–12	25–35	60–69
Age	7.94 (0.43)	11.75 (0.44)	29.53 (3.23)	64.41 (2.87)
Years of education	2.38 (0.79)	6.06 (0.24)	15.25 (1.62)	14.47 (2.21)

## Data Availability

All data, analysis code, and research materials are available upon a reasonable to the corresponding author. The data are not publicly available due to potential influence on further research projects.
